# Computational Insights into the Structural and Optical Properties of Ag-Based Halide Double Perovskites

**DOI:** 10.1021/acsami.4c22290

**Published:** 2025-03-25

**Authors:** David F. Macias-Pinilla, Francesco Giannici

**Affiliations:** Dipartimento di Fisica e Chimica “Emilio Segrè”, Università di Palermo, Viale delle Scienze, I-90128 Palermo Italy

**Keywords:** halide double perovskites, Ag-based materials, nanocrystals, stability, computational methods

## Abstract

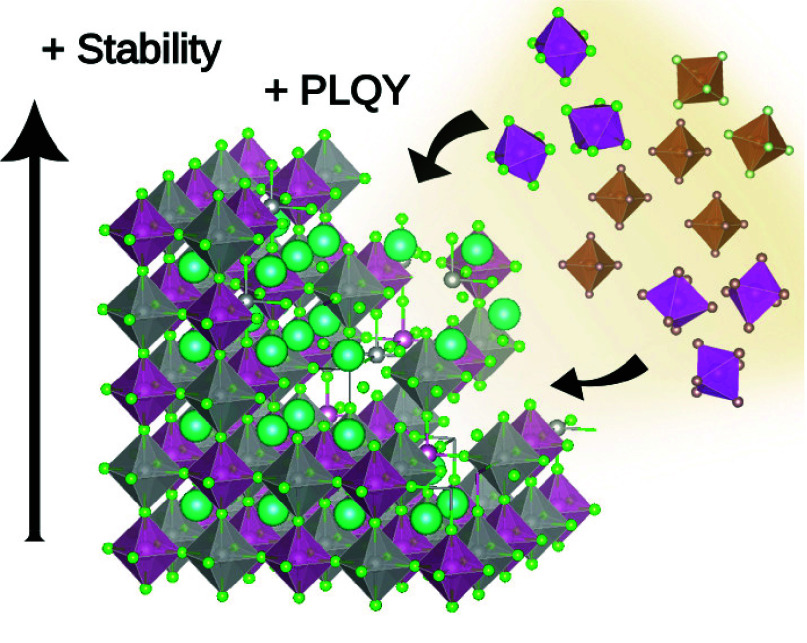

Lead-free halide double perovskites (HDP) have attracted enormous attention in recent years due to their low toxicity, excellent stability, tunable optical properties, and extensive range of compositional possibilities they present. In the very broad HDP family, Ag-based materials are of particular interest due to their easy synthesis, stability to light and moisture, and interesting optical properties, especially in the form of nanocrystals. Given the very large compositional space, theoretical studies play a crucial role in providing insights into the most promising dopant and possible defect interactions to guide the synthesis and explain the properties. In this review, we discuss recent theoretical works on Ag-based perovskites with an emphasis on density functional theory (DFT) calculations. The computational methods and tools are evaluated, assessing their relative strengths and limitations in their ability to clarify experimental results. We focus specifically on how lattice defects influence the structure and properties of HDP, including lattice and thermodynamic stability, band gap tuning, and photoluminescence.

## Introduction

Over the past decade, the field of lead halide perovskites (LHP) has seen significant advancements, particularly in optoelectronic applications such as solar cells, photodetectors, and light-emitting devices.^1−3^ The general chemical formula for these materials is *ABX*_3_, where *A* represents a monovalent cation, *B* the divalent cation Pb, and *X* a halide. This structure has paved the way to the investigation and development of many different Pb-based halides with a diverse range of properties, including high absorption in the visible light spectrum,^4,5^ tunable band gaps,^2,4^ and remarkable charge-carrier mobilities.^6^ However, the use of LHP is limited by the inherent toxicity of Pb^7^ and their instability under ambient conditions.^8^ These challenges have driven the exploration of lead-free alternatives.

The initial investigations into the search for lead-free perovskites primarily focused on substituting Pb^2+^ with isovalent cations such as Sn^2+^ or Ge^2+^, but these materials often suffer from rapid oxidation and generally poor stability.^9,10^ A promising alternative involves replacing Pb^2+^ with a monovalent cation and a trivalent cation, resulting in the ordered halide double perovskite (HDP) structure known as the elpasolite. HDP have general formula *A*_2_*B*′*B*″*X*_6_, where *A* is a monovalent cation, *B*′ and *B*″ are alternating monovalent and trivalent cations in the *B*-site, and X represents halide anions such as Cl, Br, or I. The HDP therefore maintains the overall perovskite structure while allowing for the incorporation of other elements less toxic than lead.^5,10,11^ Still displaying relatively lower power conversion efficiencies (PCEs) compared to Pb-based perovskites, these materials exhibit much higher environmental stability, and a significantly lower toxicity.^12−14^

Among HDP, those with Ag as a monovalent cation, Cs_2_Ag*B*″*X*_6_, have been the subject of extensive and ongoing research in the recent literature. They have emerged as promising candidates due to their high stability and long carrier lifetimes.^10−13,15^ These HDP all show a cubic perovskite arrangement, with [Ag*X*_6_] and [*B*″*X*_6_] octahedra connected at the corners, but the structural and optical properties can be controlled by varying the composition of the trivalent cation and anion sites. An example of such control concerns the direct or indirect character of the transitions, which can be altered by substituting the trivalent cation.^14,16^ Additionally, Ag-based HDP can achieve excellent photoluminescence properties and improved quantum yields through Na^+^ alloying and Bi^3+^ doping.^17^ These examples show the significant potential of this class of HDP for a range of prospected optoelectronic applications, including solar cells, photodetectors, and light-emitting diodes (LEDs).^11−13^

Computational methods have played a crucial role in understanding and optimizing the properties of HDP in recent years. These computational methods have been essential for studying the extensive family of HDP, employing tolerance factors and first-principles calculations based on density functional theory (DFT), which have facilitated the systematic evaluation of structural, electronic, and transport properties. Through DFT calculations, valuable information has been collected, and databases have been generated containing data about optical absorption, conversion efficiency, phonon stability, and parameters such as energy above the hull, band gaps, and electron–hole effective masses. These resources facilitate the screening and identification of promising HDP candidates for photovoltaic applications.^18^ Among the most promising candidates identified through theoretical calculations, those containing Ag have been particularly recognized for their thermodynamic stability, high defect tolerance, and optimal properties as solar absorbers.^18−23^

The synergy between theoretical and experimental research is essential to address the remaining challenges and unlock the full potential of HDP in any optoelectronic application. Currently, a wide variety of competing DFT softwares are available, each with its own implementations, strengths, and limitations, depending on the system being studied. For HDP systems, the most widely used software packages are the Vienna Ab-initio Simulation Package (VASP),^24^ ABINIT,^25^ WIEN2k,^26^ the Cambridge Serial Total Energy Package (CASTEP),^27^ and Quantum Espresso (QE).^28^ Due to such diversity, the prediction of HDP electronic properties may vary between studies, and a proper framing of the different approaches is necessary in order to rationalize their results.

In general, the first difference when the electronic properties of materials with DFT are evaluated arises from the choice of the exchange-correlation (XC) functional. This must be guided by a balance between acceptable computational cost and the required level of accuracy. Less computationally demanding functionals, such as local density approximation (LDA),^29^ almost always underestimate the band gap, but they also offer a general overview of the behavior of a given structure which can often be acceptable. Similarly, Generalized Gradient Approximation (GGA) functionals^30^ tend to yield underestimated band gap values, yet they provide a more accurate representation of the band structure. Hybrid functionals^31^ eventually give band structures that are very close to experimental observations by inserting a fraction of exact exchange from the Hartree–Fock theory, although this approach incurs a significantly higher computational expense and a somewhat arbitrary choice of the exact exchange mixing. Other methods alongside DFT, such as Many-Body Perturbation Theory with the GW approach,^32,33^ have also been employed for the study of the electronic properties of HDP, providing very accurate results, but their computational cost is significantly higher.

In this review, we summarize recent progress in the modeling and understanding of the chemical and photophysical properties of Ag-based HDP using *ab initio* techniques. The results discussed here were obtained with several different DFT software packages and XC functionals. Differences, similarities, and their alignment with experimental data are all analyzed to evaluate their accuracy in modeling HDP. We focus on outputs related to composition, doping, and vacancies concerning structural stability and electronic characteristics. Additionally, we highlight the current challenges and future opportunities in the field, aiming to provide a comprehensive understanding of DFT methods in studying systems such as double perovskites for advancing sustainable and efficient optoelectronic technologies.

## Crystal Structure and Stability

### Bulk Structure

**Figure 1:**
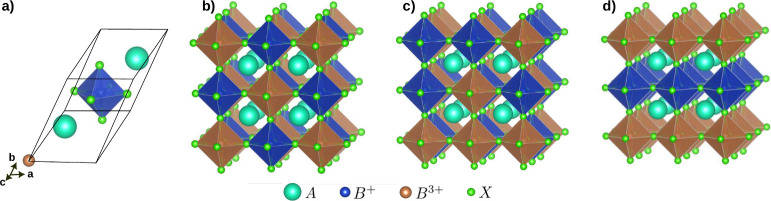
(a) HDP fcc primitive unit cell. Possible arrangements of monovalent and trivalent cations in the B-site of a HDP: (b) ordered (elpasolite); (c) disordered (one of many different arrangements is shown); (d) layered.

HDP with formula *A*_2_*B*′*B*″*X*_6_ usually feature an alternating arrangement of corner-sharing *B*″*X*_6_ and *B*′*X*_6_ octahedra. These structures adopt a face-centered cubic (fcc) arrangement within space group *Fm*3̅*m* (No. 225), whose primitive cell is depicted in Figure 1a. In this structure, the *A* atoms are placed at Wyckoff position 8c, *B*′ atoms at 4b, *B*″ atoms at 4a, and *X* atoms occupy the 24e position. The alternating arrangement of octahedra in the two different *B*-sites can be perfectly ordered (Figure 1b) as in the mineral elpasolite, randomly distributed (with a space group *Pm*3̅*m* resembling a simple perovskite solid solution, Figure 1c), or layered (Figure 1d), although the latter is rarely observed in HDP. As is discussed in the following sections, the degree of order at the B-site plays a crucial role in determining the optical properties of HDP. For simulations of the elpasolite structure, a small unit cell with 10 atoms can be defined for the elpasolite ordered arrangement (Figure 1a). For disordered or layered structures, the unit cell increases to 40 atoms or more, making the computational study more demanding. This also applies to the study of doping, alloying, or other point defects, as the supercell size increases significantly.

Among all known double perovskites crystallizing in the space group Fm3̅m at room temperature (regardless of thermodynamic stability toward decomposition) we can find combinations of 7 elements as *A*-site cations, 8 as *B*′-site cations, 34 as *B*″ cations, and 5 as *X*-site anions, leading to approximately 9,520 possible combinations.^5^ This number is even higher when considering possible modifications due to alloying, doping, or vacancy defects given that each of these often changes the properties of the HDP by a great extent. Thanks to DFT methods and machine learning approaches,^34,35^ the selection of materials can in principle be narrowed down to those with the best properties. Within the most promising compositions, those based on Ag were identified almost one decade ago. These exhibit a wide range of band gaps, from 1.57 to 3.33 eV,^36−39^ with values suitable for various optoelectronic applications. Furthermore, Ag-based perovskites demonstrate excellent stability under ambient conditions of heat and moisture, along with good mechanical stability.^40^ Thanks to their relatively easy synthesis, they can be produced in the form of large single crystals,^41^ nanocrystals,^42,43^ and films.^44,45^ However, significant efforts are still required to address the low photoluminescence reported in these materials, which limits their practical applications.^5,11,13^

### Stability

To evaluate theoretically the stability of HDP materials, both crystallographic and thermodynamic considerations are commonly used. Crystallographic factors are important in the early stages of material discovery, as they enable the rapid identification of materials that, based on their atomic size and radii, are geometrically plausible for forming the elpasolite structure, narrowing the material search space. However, their accuracy is limited as they do not account for any electronic effect beyond simple hard sphere packing of ions. In this regard, thermodynamic considerations provide a more comprehensive understanding of the stability. In the following sections, we describe the crystallographic stability of Ag-HDP materials and further complement these studies with thermodynamic analyses, including formation energies and decomposition enthalpies, to provide a more detailed characterization of HDP stability.

#### Crystallographic Stability

Crystallographic stability is typically analyzed based on Goldschmidt tolerance factor (*t*), the octahedral factor (μ), and a recently proposed new factor, τ,^40,46^ that takes into account the oxidation states of the *A* cation (*n*_A_). These stability factors are defined respectively as
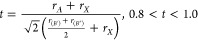
1
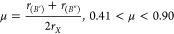
2
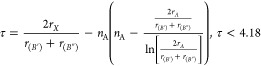
3with *r*_*A*_, *r*_(*B*′)_, , and *r*_*X*_ being the ionic radius of the corresponding HPD cations and anions. Normally, Shannon ionic radii are used in these calculations. The corresponding limits for stable HDP are reported alongside the respective eqs 1–3. The tolerance factors described above are an adaptation of the factors proposed for simple perovskites. By a simple averaging the ionic radii *r*_(*B*′)_ and , the corresponding term for the ionic radius *r*_*B*_ in the simple perovskite is replaced. Of course, this can present an inaccurate description for the case of disordered HDP. Due to a nonhomogeneous distribution of *B*′*X*_6_–*B*″*X*_6_ octahedra, a simple average of ionic radii may be insufficient in describing the local deviations from the average crystal structure. Additionally, these factors are limited in their application when considering doping or vacancy defects, whose effect might be either localized or spread through a larger volume. In the case of alloys, as combinations of different cations or anions, averages of atomic radii can be considered. For example, in ref (20), the average ionic radius *r*_*X*_ for Cs_2_AgSb(Br_6–*x*_Cl_*x*_) is taken simply as *r*_*X*_ = {[(6 – *x*)*r*_Br_ + *xr*_Cl_]/6}, (0 < *x* < 6). This suggests a linear relationship between the limits of pure chlorine and pure bromine in terms of the crystallographic stability factors, assuming the so-called Vegard law is valid (i.e., the steric effect of alloying is spread throughout the lattice).

**Figure 2:**
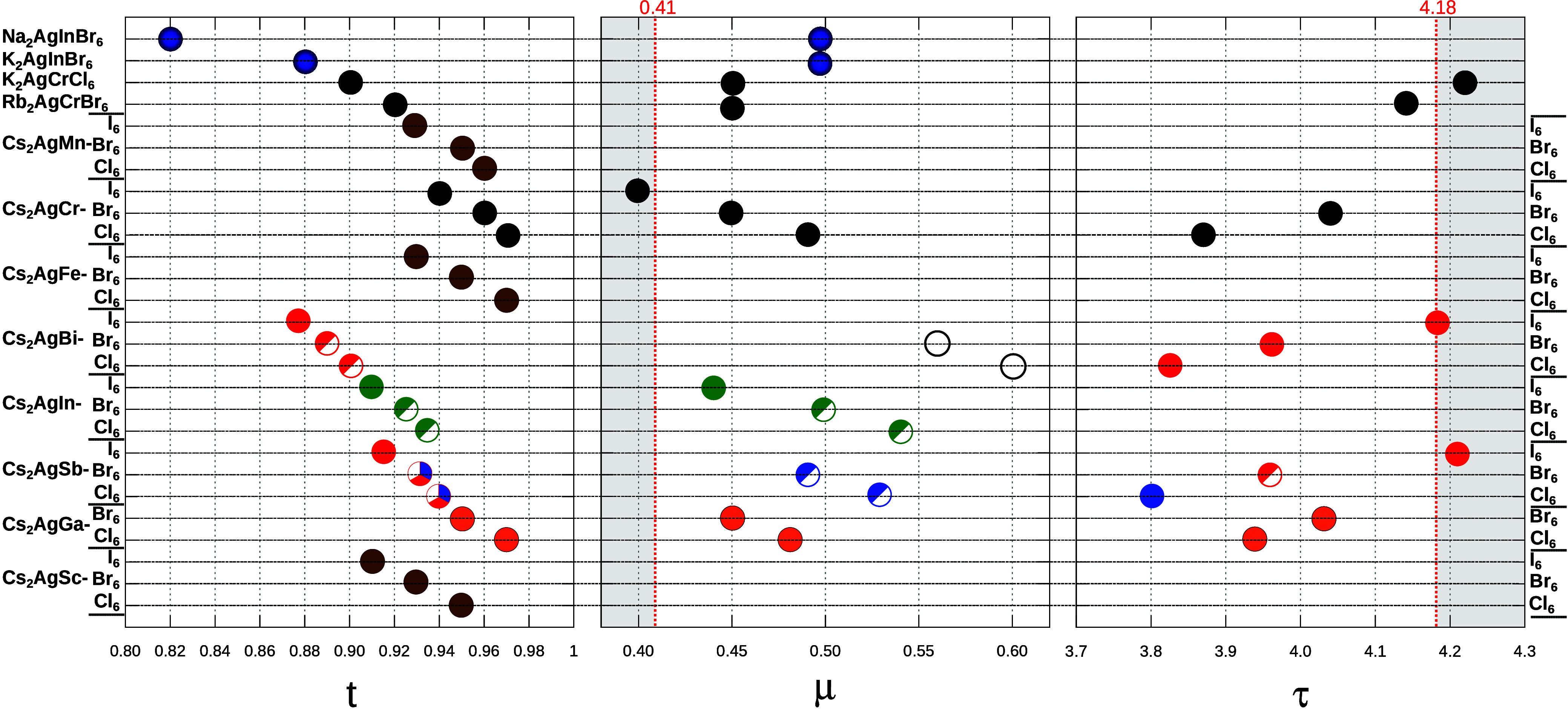
Reported stability from (a) t-factor, (b) octahedral μ-factor, and (c) τ-factor for Ag-based HDP. The gray areas fall outside the stability limits. Taken from refs (40) (blue), (47) (white), (48) (red), (39) (green), (49) (black), (50) (dark blue), (51) (brown) and (52), and (53) (orange). Some missing values were not found in the literature.

Figure 2 presents a summary of the crystallographic factors reported for Ag-based HDP. All perovskites fall within the stability limits defined by the *t*-factor. As the ionic radius *r*_*X*_ increases, the *t*-factor deviates further from the ideal value of *t* = 1 (ideal cubic structure). This is evident when comparing the perovskites to Cl, Br, and I in Figure 2a. As the ionic size increases down the halide group, the *t*-factor is higher for Cl compounds and lower for I compounds. The values of the octahedral factor (μ) are shown in Figure 2b. All of the HDP fall within the stability limits except for Cs_2_AgCrI_6_, suggesting its thermodynamic instability and the difficulty of its possible synthesis. Several Ag-HDP predicted stable by both the *t* and octahedral factors have, however, been found to be unstable, presenting significant experimental challenges, as is generally the case of iodide HDP.^54,55^ The main challenge associated with iodide is its large atomic radius (2.2 Å), which requires selecting a suitable trivalent cation to fulfill the criteria of the tolerance factor (*t*) and (μ) in order to form a stable HDP. With an appropriate combination of the tolerance factor and the radius ratio of trivalent cations, some iodine-based double perovskites have been theoretically proposed to be stable.^54^ Several of these materials, including Cs_2_Na(Ce, Nd, Gd, Tb, Dy)I_6_^56^ and Cs_2_Ti(Br,I)_6_,^57^ have recently been synthesized experimentally, thereby expanding the family of HDP and opening new directions for scientific investigation.

Factors such as τ are, therefore, being explored to improve stability predictions. Indeed, the τ factor predicts the instability of compounds with I, as shown in Figure 2c. Overall, the geometrical stability factors *t*, μ, and τ may provide a first general assessment of the stability of HDP compositions in terms of their proximity to the perovskite aristotype. These crystallographic factors provide a useful first-pass material screening, after which it is necessary to analyze the electronic interactions through thermodynamic factors to more accurately predict the stability of HDP.

#### Thermodynamic Stability

The thermodynamic stability of HDP can be evaluated with DFT through total energy calculations in terms of formation energy and decomposition enthalpy. The formation energy measures the energy released or absorbed when the compound is formed from its reactants; the more negative its value, the more exothermic the formation and the greater the HDP stability (the opposite is true for positive energies). In the case of pristine HDP, formation energy is generally calculated with respect to the atoms in a pure solid substance as
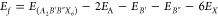
4where  is the total energy of the HDP and *E*_*A*_, *E*_*B*′_, *E*_*B*^″^_, *E*_*X*_ are the corresponding energies of isolated atoms maintaining the stoichiometry.^20,52,55,58−60^ The formation energy, depending on the synthetic pathway, can also be calculated in terms of precursor compounds and defined as the energy difference between the HDP and its halide precursors.

To assess the relative ease of formation of points defects and alloying in a charge state *q*, the formation energy is also used, defined as
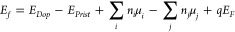
5where *E*_*Dop*_ and *E*_*Prist*_ are the total energies of the defective and pristine supercells, *n*_*i*_ and *n*_*j*_ are the number of atoms removed and added respectively, μ_*i*_ and μ_*j*_ are the corresponding chemical potentials, and *E*_*F*_ is the Fermi energy.^61−63^

In the literature, the projector augmented wave (PAW) method^64^ with the GGA^65^ functionals PBE and PBEsol^30^ are commonly used for thermodynamic stability calculations. These functionals have been shown to be efficient and provide results in agreement, in terms of formation energy and decomposition enthalpy, with more computationally demanding functionals such as hybrid functionals.^21,48,66^Table 1 presents the formation energies for various Ag-based HDP, calculated using different DFT packages, i.e., QE, VASP, and Wien2k. Whenever different packages were used, only very small discrepancies were found, up to about 0.02 eV/atom. These small discrepancies are present even when using the same software and functional. They can be attributed to the initial arrangement of atomic positions for subsequent atomic relaxation, force and energy convergence criteria, the number of *k*-points evaluated, and cutoff energies and densities for the plane waves. All these parameters influence the determination of total energies in systems through DFT, making the evaluation of their convergence essential for obtaining accurate results.

**Table 1:** Formation Energy of Ag-Based HDP with Respect to Pure Elements

HDP	Formation Energy (eV/atom)	Functional	Code	Ref.
Cs_2_AgInBr_6_	–1.110	PBE	VASP	(52)
Cs_2_AgSbBr_6_	–1.080	PBE	VASP	(52)
	–1.095	PBE	QE	(58)
	–1.107	PBE	VASP	(20)
Cs_2_AgBiBr_6_	–1.13	PBE	VASP	(52)
	–1.136	PBE	VASP	(55)
	–1.156	PBE	QE	(58)
Cs_2_AgBiCl_6_	–1.370	PBE	QE	(58)
Cs_2_AgSbCl_6_	–1.308	PBE	QE	(58)
	–1.304	PBE	VASP	(20)
Cs_2_AgBiI_6_	–0.885	PBE	QE	(58)
Cs_2_AgSbI_6_	–0.827	PBE	QE	(58)
Cs_2_AgCrCl_6_	–1.000	PBEsol	Wien2K	(59)
Cs_2_AgCrBr_6_	–1.410	PBEsol	Wien2K	(59)
Cs_2_AgCrI_6_	–1.410	PBEsol	Wien2K	(59)
Cs_2_AgScCl_6_	–1.805	PBE	VASP	(51)
Cs_2_AgScBr_6_	–1.539	PBE	VASP	(51)
Cs_2_AgScI_6_	–1.191	PBE	VASP	(51)
Cs_2_AgFeCl_6_	–1.205	PBE	VASP	(51)
Cs_2_AgFeBr_6_	–0.983	PBE	VASP	(51)
Cs_2_AgFeI_6_	–0.690	PBE	VASP	(51)
Cs_2_AgGaBr_6_	–1.080	PBE	VASP	(52)

According to Table 1, all HDP have negative formation energies, indicating their thermodynamic stability with respect to the elements. However, some iodine HDP have higher energies, which suggests lower stability compared with the other halides. This is consistent with the tolerance factor, τ, which deviates more from unity in iodides. It is worth noting that the perovskites with the greatest stability – i.e. lowest formation energy, are those containing CrBr and CrI. Nonetheless, these types of perovskites have only been proposed theoretically, and their synthesis has yet to be reported. This suggests the need for a more comprehensive analysis, not only in terms of formation energy but also considering other properties such as decomposition enthalpies and electronic stability. HPD such as Cs_2_AgBiBr_6_,^67−69^ alloys of Cs_2_AgInBr_6_, Cs_2_AgSbBr_6_,^70^ Cs_2_AgBiCl_6_,^71^ Cs_2_AgSbCl_6_,^15,38,72,73^ Cs_2_AgFe(Cl,Br)_6_,^74,75^ and rare earth materials as Cs_2_AgScCl_6_^76^ have been reported experimentally confirming the predictions made by the formation energy calculations. It is worth also mentioning that Cs_2_AgInCl_6_^11^ was successfully synthesized in several studies, but its formation energy is not reported here.

Another measure of the stability of HDP is the decomposition enthalpy Δ*H*, defined as the difference between the total energy of the decomposed products [*E*(product)] and the total energy of the HDP:^21,40,48,55,58,62^
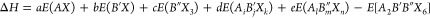
6

Since HDP contains multiple cations, it exhibits a wide range of decomposition pathways into binary and ternary compounds. In eq 6, parameters *a*–*e* and *i*–*n* depend on the specific stoichiometry of the ternary compounds. The enthalpy of decomposition can be calculated either for binary compounds alone (with *d* = *e* = 0) or for both binary and ternary compounds. The more positive the decomposition enthalpy, the lower the probability of decomposition; the opposite occurs for negative values. According to the literature, the most probable decomposition pathways of Ag-based HDP are
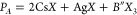
7
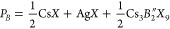
8
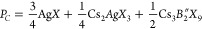
9
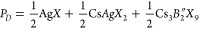
10

Pathway *P*_*A*_ is the complete decomposition to the simplest binary compounds only, while *P*_*B*_ also involves the formation to the most stable ternary compound. Since Cs–Ag-X compounds also exist, their formation is accounted for in pathways *P*_*C*_ and *P*_*D*_. In Tables 2–4, we present the decomposition enthalpies, the corresponding decomposition pathways, and the code used.

**Table 2:** Decomposition Enthalpy of AgBr-Based HDP^a^

HDP	Pathway	Δ*H* (meV/atom)	Code	Ref.
Cs_2_AgBiBr_6_	*P*_*A*_	64, 42, 66.5	VASP	(55, 52, 21)
		41.9	QE	(58)
	*P*_*B*_	24, 30, 14.8	VASP	(55, 52, 21)
		3.9	QE	(58)
	*P*_*C*_	16, 5.0	VASP	(55, 21)
		0.1	QE	(58)
	*P*_*D*_	11, 3.9	VASP	(55, 21)
		1.1	QE	(58)
Cs_2_AgSbBr_6_	*P*_*A*_	62	VASP	(52)
		54.7	QE	(58)
	*P*_*B*_	–30	VASP	(52)
		–3.9	QE	(58)
	*P*_*C*_	–7.7	QE	(58)
	*P*_*D*_	–6.7	QE	(58)
Cs_2_AgInBr_6_	*P*_*A*_	30, 55.8	VASP	(52, 21)
	*P*_*B*_	22, 0.3	VASP	(52, 21)
	*P*_*C*_	–9.5	VASP	(21)
	*P*_*D*_	–10.5	VASP	(21)
Cs_2_AgGaBr_6_	*P*_*A*_	–18	VASP	(52)
	*P*_*B*_	9	VASP	(52)

a References are listed in the same order as the respective decomposition enthalpy values. All decomposition enthalpies were calculated with the PBE functional.

Inspecting the decomposition enthalpy to halides in Tables 2–4, it can be seen that for most Ag-based HDP, the decomposition enthalpy toward the binary compounds alone is higher compared to the other decomposition pathways. Except for the iodide and Ga perovskites, all values for the binary decomposition enthalpy are on the order of tens of meV/atom, sometimes reaching hundreds (Tables 2–3), indicating that it is unlikely for the HDP to decompose into binary compounds. In the case of perovskites with iodine (Table 4), although they have positive decomposition enthalpies for binary decomposition, their values are very small. In fact, they exhibit negative decomposition enthalpies for all other decomposition pathways, showing lower stability compared with other Ag-based perovskites. This confirms the results obtained from the formation energy (see Table 1), τ factor, and the difficulties in achieving their synthesis.

**Table 3:** Decomposition Enthalpy of AgCl-Based HDP^a^

HDP	Pathway	Δ*H*(meV/atom)	Code	Ref.
Cs_2_AgBiCl_6_	*P*_*A*_	100	QE	(58)
		121.3	VASP	(21)
	*P*_*B*_	21.7	QE	(58)
		26.7	VASP	(21)
	*P*_*C*_	16	QE	(58)
		16.1	VASP	(21)
	*P*_*D*_	14.7	QE	(58)
		14.6	VASP	(21)
Cs_2_AgSbCl_6_	*P*_*A*_	75.9	QE	(58)
		94.3	VASP	(21)
	*P*_*B*_	11.6	QE	(58)
		15.9	VASP	(21)
	*P*_*C*_	5.9	QE	(58)
		5.3	VASP	(21)
	*P*_*D*_	4.5	QE	(58)
		3.8	VASP	(21)
Cs_2_AgInCl_6_	*P*_*A*_	88.8	VASP	(21)
	*P*_*B*_	18.6	VASP	(21)
	*P*_*C*_	8.1	VASP	(21)
	*P*_*D*_	6.6	VASP	(21)

a All decomposition enthalpies were calculated with the PBE functional.

**Table 4:** Decomposition Enthalpy of AgI-Based HDP^a^

HDP	Pathway	Δ*H* (meV/atom)	Code	Ref.
Cs_2_AgBiI_6_	*P*_*A*_	1.2	QE	(58)
		9.5	VASP	(21)
	*P*_*B*_	–22	QE	(58)
		–22.8	VASP	(21)
	*P*_*C*_	–23.9	QE	(58)
		–28.1	VASP	(21)
	*P*_*D*_	–19.1	QE	(58)
Cs_2_AgSbI_6_	*P*_*A*_	15.7	VASP	(21)
	*P*_*B*_	–22.5	VASP	(21)
	*P*_*C*_	–27.8	VASP	(21)
Cs_2_AgInI_6_	*P*_*A*_	2.6	VASP	(21)
	Cs_2_AgI_3_ + InI_3_	–18.6	VASP	(21)
	CsI + AgI + CsInI_4_	–43.1	VASP	(21)

a The different pathways for Cs_2_AgInI_6_ presented were shown to be the most probable decomposition pathways for this HDP. All decomposition enthalpies were calculated with the PBE functional.

When considering the decomposition energy into ternary compounds (pathways *P*_*B*,*C*,*D*_), the decomposition enthalpy generally decreases for all Ag-based HDP, with some even reaching negative values. This suggests that the perovskite’s stability is compromised, leading to a tendency for decomposition into ternary compounds. Therefore, theoretical works must account for all possible decomposition pathways into binary and ternary compounds to provide an accurate estimation of HDP stability. The perovskites that exhibit the greatest theoretical stability are Cs_2_AgBiBr_6_ (Table 2) and all those with Cl (Table 3). Although their decomposition enthalpy is low for some pathways toward ternary compounds, all the values are positive. This fact demonstrates the agreement between theory and experiment, as these perovskites have been synthesized and continue to be extensively studied.

Tables 2–4 show some discrepancies in the decomposition enthalpy values obtained depending on whether the same or different software was used. As mentioned earlier, this can be attributed to slight changes in the input parameters during the execution of the DFT calculations. Despite the discrepancies reaching up to 26 meV/atom in some cases, the positive/negative decomposition enthalpy values remain consistent, which provides insight into whether the material is likely to decompose or remain stable. Clearly, larger absolute values indicate a higher probability of decomposition or stability depending on the sign of the enthalpy. Additionally, the enthalpy values show similar trends when changing the decomposition pathway, when comparing the software packages. Although the results presented are reported by only a few software packages, the trends shown in both the formation energy and decomposition enthalpy exhibit a considerable degree of agreement. It should also be noted that these theoretical results align with experimental findings regarding stability. This validates and motivates the use of DFT methods for predicting the potentially most stable perovskites that can be synthesized.

We must emphasize that to fully assess the HDP thermodynamic stability, it is essential to include, alongside formation energy and decomposition enthalpy, the energy above the convex hull (*E*_*hull*_). *E*_*hull*_ is an accurate indicator of a compound’s stability, as it is determined by evaluating all possible phase decomposition reactions of the compound.^77,78^ While *E*_*hull*_ can be calculated using DFT methods, the extensive number of phase decomposition reactions among competing compounds makes its computation highly demanding. However, with recent technological advancements, methodologies such as machine learning (ML) are now being employed to study this type of thermodynamic property in materials. ML algorithms trained with outputs from DFT calculations have shown agreement with both the *E*_*hull*_ values and the experimentally observed stabilities in HDP, and consequently, in Ag-based HDP.^77,78^ Moreover, ML algorithms have been used to propose new materials as potential stable candidates for synthesis and subsequent optoelectronic applications. ML methods, combined with DFT training sets, have made significant progress in predicting electronic and thermodynamic properties of perovskites and are emerging as valuable auxiliary tools to advance computational and experimental materials science together.^34,79^

### Doping, Alloying and Point Defects

**Figure 3:**
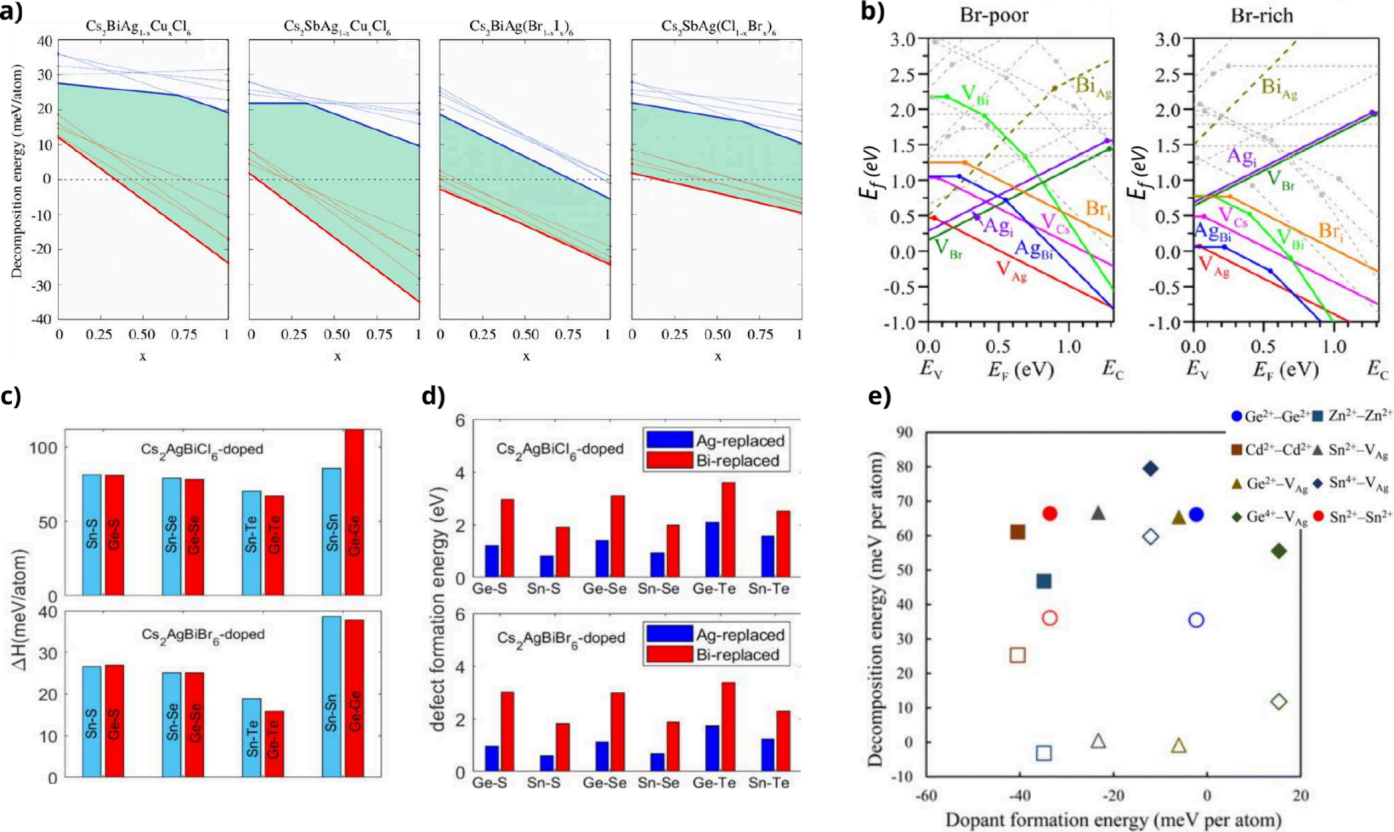
(a) Decomposition energy as a function of doping concentration “*x*” for Cs_2_BiAg_1–*x*_Cu_*x*_Cl_6_, Cs_2_SbAg_1–*x*_Cu_*x*_Cl_6_, Cs_2_BiAg(Br_1–*x*_I_*x*_)_6_, and Cs_2_SbAg(Cl_1–*x*_Br_*x*_)_6_. (b) Formation energy of points defects in Cs_2_AgBiBr_6_ as a function of the Fermi energy (*E*_*f*_) under Br-poor (left) and Br-rich (right) conditions. (c) Decomposition enthalpy of doped Cs_2_AgBiBr_6_ and Cs_2_AgBiCl_6_ and (d) defect formation energy. (e) Relation between stability and dopant formation energy in divalent-doped Cs_2_AgSbCl_6_ perovskite. Filled symbols and hollow symbols represent the decomposition toward binary compounds and binary + ternary compounds, respectively. (a) Reprinted with permission from ref (58), Copyright 2018, American Chemical Society. (b) Adapted with permission from ref (55), Copyright 2016 Wiley-VCH Verlag GmbH & Co. KGaA, Weinheim. (c and d) Adapted with permission from ref (80), Copyright 2021, American Chemical Society. (e) Adapted from ref (62) with permission from the Royal Society of Chemistry, Copyright 2022

To enhance the stability of perovskites or to control their optical properties, theoretical and experimental methods were employed to explore chemical mixing at the cation and/or anion sites in HDP. Given the feasibility of studying perovskite stability from a theoretical perspective, several studies have proposed that specific configurations with alloying, atomic site changes, and doping result in enhanced stability and optical properties. In ref (58), the decomposition energies for Cs_2_BiAg_1–*x*_Cu_*x*_Cl_6_, Cs_2_SbAg_1–*x*_Cu_*x*_Cl_6_, Cs_2_BiAg(Br_1–*x*_I_*x*_)_6_, and Cs_2_SbAg(Cl_1–*x*_Br_*x*_)_6_ (0 < *x* < 1) were studied, as shown in Figure 3a. Thin lines represent the decomposition energies for various reaction pathways (including binary and ternary compounds), using both LDA^29^ (blue lines) and PBE functionals (red lines). The thick lines indicate the lowest decomposition energy pathway at each concentration “*x*”. The results obtained through the PBE show a decreasing trend with a constant rate of change as the concentration “*x*” increases. The LDA exhibits some variations in the rate of change, and in certain cases, a majority of the values show positive enthalpy. However, it preserves the trend of decreasing stability as the concentration *x* increases, consistently with PBE. Having mentioned that PBE shows good agreement with experimental results, it may also be added that to some extent the LDA functional reproduces correct trends for the stability of HDP, while giving less reliable total energies. In Figure 3a, the increase in the Cu concentration causes a decrease in the stability of the perovskites. This may be a consequence of Cu preference for 4-fold coordination over 6-fold coordination in the perovskite octahedra, thereby allowing decomposition into ternary compounds.^21^ As seen in Tables 2 and 4, perovskites with Br exhibit superior stability compared to those with I. Therefore, when Br/I is mixed, a decrease in stability is observed as the concentration of I increases. In the case of Cl/Br mixing, lower concentrations of Br maintain positive decomposition enthalpy values, but as the Br percentage increases, system stability decreases. All of these observations align with the decomposition enthalpy values in Tables 2–4, where Cl-based perovskites generally exhibit greater stability compared to those with Br or I, with I-based perovskites being the least stable.

Xiao et al., in ref (55), conducted a study on 20 point defects in Cs_2_AgBiBr_6_ under Br-rich and Br-poor conditions. They investigated four vacancies (V_Cs_, V_Ag_, V_Bi_, and V_Br_), four interstitials (Cs_*i*_, Ag_*i*_, Bi_*i*_, and Br_*i*_), six cation-on-cation antisites (Cs_*Ag*_, Cs_*Bi*_, Ag_*Cs*_, Ag_*Bi*_, Bi_*Cs*_, and Bi_*Ag*_), three cation-on-anion antisites (Cs_*Br*_, Ag_*Br*_, and Bi_*Br*_), and three anion-on-cation antisites (Br_*Cs*_, Br_*Ag*_, and Br_*Bi*_) as a function of the Fermi level (see Figure 3b). Ag vacancies were found to be particularly prone to be generated due to their low formation energy under both Br-rich and Br-poor conditions. Compared to the high defect tolerance of lead-based perovskites, which contain only a divalent cation, and where defects typically manifest as shallow states, located near the valence band maximum (VBM) or conduction band minimum (CBM) with negligible impact on optical properties,^13^ HDP exhibit substantial differences due to the presence of two distinct *B*-site cations, leading to the formation of additional antisite defects.^81^ DFT calculations of thermodynamic transition energy levels were used to understand the role of defects in Ag-HDP. The most easily formed defects are Ag vacancies, which act as shallow acceptors, while Ag interstitials behave as shallow donors. In contrast, *B*‴ vacancies and Ag/*B*‴ antisite defects are deep acceptors and become dominant under Br- or Cl-rich conditions. Although HDP exhibit lower defect tolerance compared to lead-based perovskites, synthesis techniques with controlled growth conditions can suppress deep defects under halogen- and *B*‴-poor or -rich environments, enabling optoelectronic applicability.^55,82^

In ref (80), the decomposition and defect formation energies of Cs_2_AgBiCl_6_ and Cs_2_AgBiBr_6_ perovskites doped with M^2+^ ions from group IV (Sn^2+^ and Ge^2+^) and L^2–^ ions from group VI (S^2–^, Se^2–^, and Te^2–^), at a 25% concentration were studied. To calculate stability in terms of decomposition enthalpy toward binary compounds, two doping strategies were considered. First, two M^2+^ ions replace a monovalent cation Ag^+^ and a trivalent cation Bi^3+^ (M^2+^–M^2+^ doping), and the second strategy involves an M^2+^-L^2–^ pair replacing an Ag–(Cl–Br) pair, maintaining charge balance (Figure 3c). To calculate the defect formation energy, the replacement of a Ag site and a Bi site was studied (Figure 3d). One M^2+^–L^2–^ pair then replaces either a Ag–Cl,Br pair (labeled Ag-replaced) or a Bi–Cl,Br pair (labeled Bi-replaced). Figure 3c shows that the decomposition energies into binary compounds are positive and typically exceed 20 meV/atom, suggesting the potential stability of the doped systems. Nevertheless, further studies on decomposition toward ternary compounds are needed to establish stability under this type of doping. Figure 3d shows positive formation energies, indicating the instability introduced by doping. However, the formation energies are lower when Ag is replaced, implying that potential dopants might be more likely to substitute at the Ag site rather than the Bi site.

The doping with divalent cations in Cs_2_AgSbCl_6_ perovskite has also been studied in ref (62). Following the same strategy as before and to maintain charge balance, two divalent cations replace a monovalent cation and a trivalent cation, with doping percentages of 25%. Furthermore, charge compensation with Ag vacancies was also considered. Figure 3e shows the formation energy and decomposition energy toward binary compounds and Cs_2_Sb_3_Cl_9_ plus binary compounds, represented by filled and hollow symbols, respectively. Among the divalent dopant cations, Cd^2+^ and Zn^2+^ exhibit low formation energy and positive decomposition energy, indicating the possibility of incorporating this type of doping into the pristine double perovskite.

All in all, we have seen how various DFT methods provide insights into how doping, defects, and alloying affect the stability of double perovskites. Some of them explain why synthesis difficulties arise in certain materials, while others propose new doping strategies that could potentially be applied to future syntheses. Continued refinement of computational models, including the improvement of exchange-correlation functionals and the integration of machine learning techniques, along with the optimization of computational resources through GPU parallelization, is necessary for exploring various compositions to optimize these materials for practical applications. It is important to mention that for a more comprehensive understanding of the stability and synthesis challenges of HDP, an analysis of dynamic stability with respect to temperature, pressure, and deformation should be conducted. Although this particular aspect falls outside the scope of the review, we highlight that dynamical stability has been extensively studied for simple perovskites, but it remains largely unexplored for HDP.

### Electronic Structure and Optical Properties

Contrary to expectations, the electronic properties of HDP differ significantly from those of the simple perovskites *ABX*_3_. While simple perovskites typically exhibit relatively small direct band gaps, double perovskites often have large indirect band gaps or direct parity-forbidden band gaps with low oscillator strength. Currently, these challenges are being addressed by exploring changes in composition, doping, or alloying. In the following sections, we present the most relevant theoretical results regarding the electronic properties of HDP, and the proposed methods to improve their characteristics as a guide for the design of high-performance HDP.

### Band Structure: General Features

**Figure 4:**
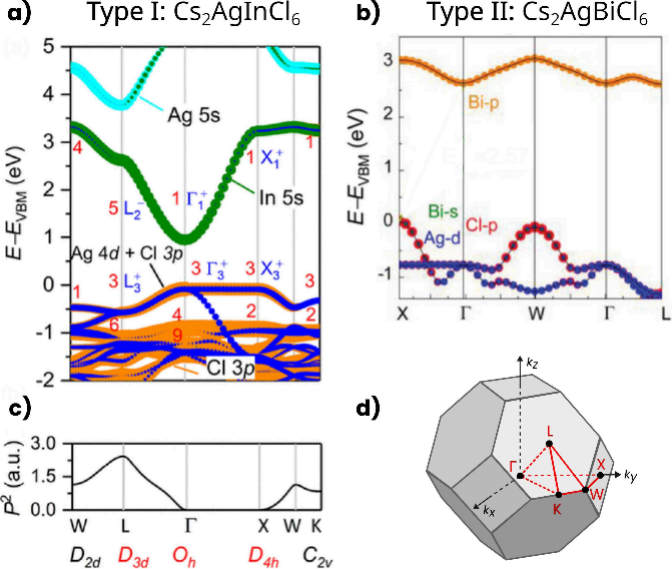
Electronic bands and states of (a) Type I and (b) Type II HDP. (c) Transition probability between the VBM and CBM for Cs_2_AgInCl_6_, with zero probability at the Γ point. (d) First Brillouin zone of the fcc lattice with high symmetry points and paths. (a and c) Reprinted with permission from ref (83), Copyright 2017, American Chemical Society. (b) Adapted with permission from ref (16), Copyright 2019 Wiley-VCH Verlag GmbH & Co. KGaA, Weinheim.

Ag-based HDP can be classified into two categories based on their optical properties depending on lone-pair states (s^0^).^16^ Type I perovskites have a direct bandgap at the Γ point of the Brillouin zone (see Figure 4a, 4d). For example, in Cs_2_AgIn*X*_6_, the VBM is formed by cationic Ag-4d states and anion *X*-p states, while the CBM is due to antibonding states formed by 5s orbitals of In and Ag, and *X*-p orbitals. The CBM, which is primarily composed of delocalized In-5s states, along with symmetry inversion, leads to the VBM and CBM having the same even parity, which eventually results in a parity-forbidden direct transition at the Γ point,^83^ as shown in Figure 4c.

Type II perovskites, with lone-pair s electrons (s^2^) on the *B*″ cation, exhibit indirect band gap with the VBM located at the X point and the CBM at the L point (see Figure 4b). The VBM is primarily composed of Ag-4d, *B*″-s, and *X*-p states, while the CBM is mainly formed by *B*″-p and *X*-p states.^16,83,84^ The electronic mismatch in the angular momentum of the atomic orbitals between Ag and *B*″ in the conduction and valence bands is responsible for the indirect nature of these type of perovskites.^5,85^ Despite their indirect nature, these perovskites exhibit a relatively smaller band gap between 2.0 and 3.0 eV (which can be tuned through doping or alloying), good stability against heat and moisture, and facile synthesis of crystals, nanocrystals, and films.^42,43^ All of these features have driven interest in optimizing their optical properties.

Theoretical studies indicate that all Ag-based perovskites can be classified into the two types previously described based on their electronic structure. The shapes of the calculated VBM and CBM for different Ag perovskites remain consistent, with only slight modifications. Among the Ag perovskites, main differences are found in the position of the electronic bands, resulting in different band gap values, as summarized in Table 5. Table 5 provides a comparison of the calculated and experimental band gaps for Ag-based HDP using different exchange-correlation functionals, including the pure GGA PBE and PBEsol functionals, the hybrid HSE,^103^ the meta-GGA modified Becke–Johnson (mBJ),^104^ and also the GW method,^33^ as implemented in VASP, WIEN2k, Quantum Espresso, Yambo,^105^ and CASTEP.

**Table 5:** Ag-Based Bandgaps^a^

HDP	Method	Band Gap (eV)	Ref.	Exp. (eV)
Cs_2_AgBiBr_6_ (Indirect)	HSE	1.93*^V^, 2.06 *^V^, 1.89*^V^, 1.84*^V^	(19, 37, 86, 87)	2.19^37^
		1.77*^V^	(88)	1.9^89^
	mBJ	1.94*^W^, 2.07^*W*^	(48) [•]	2.18^90^
	PBE	1.10*^W^, 1.32^*W*^, 1.18*^Q^, 1.42^*Q*^	(48) [•], (91) [•]	
		1.09*^V^	(86)	
	PBEsol	1.02*^W^, 1.25^*W*^, 1.33^*Q*^, 1.10^*Q*^	(48) [•], (91), (92)	
	G_0_W_0_	1.8*^Y^, 2.1*^Y^	(89), (93)	
Cs_2_AgBiCl_6_ (Indirect)	HSE	2.62*^V^	(19)	2.77^37^
	mBJ	2.35*^W^, 2.79^*W*^	(48) [•]	2.4^94^
	PBE	1.49*^W^, 1.82^*W*^, 1.57*^Q^, 1.91^*Q*^	(48) [•],^91^ [•]	2.2^89^
	PBEsol	1.40*^W^, 1.71^*W*^, 1.81^*Q*^, 1.71^*Q*^	(48) [•], (91, 92)	
	G_0_W_0_	2.40*^Y^	(89)	
Cs_2_AgBiI_6_ (Indirect)	HSE	1.75*^W^, 1.33^*W*^	(48) [•]	1.75^95^
	mBJ	1.30*^W^, 1.33^*W*^, 1.46^*W*^	(48) [•], (96)	
	PBE	0.67*^Q^, 0.89^*Q*^, 0.62*^W^, 0.83^*W*^	(91) [•], (48) [•]	
	PBEsol	0.83^*Q*^, 0.63*^,*W*^, 0.78^*W*^	(91), (48) [•]	
Cs_2_AgInBr_6_ (Direct)	HSE	1.47^*V*^, 1.50^*V*^, 1.64^*V*^	(52), (97, 98)	1.57^36^
	PBE	0.21^*V*^	(52)	
Cs_2_AgInCl_6_ (Direct)	HSE	2.38^*V*^, 2.32^*V*^, 2.38^*V*^, 3.0^*V*^	(83), (21), (99), (98)	3.33^39^
	PBE	1.03^*V*^, 1.00^*V*^, 1.03^*V*^	(83), (21), (99)	3.23^100^
	G_0_W_0_	3.27^*V*^	(17)	3.2^101^
Cs_2_AgSbBr_6_ (Indirect)	HSE	0.88^*C*^, 1.67*^V^, 1.46*^V^, 1.37*^V^	(102), (97), (87), (88)	1.64^87^
	mBJ	1.5*^W^, 1.54 ^*W*^	(48) [•]	
	PBE	0.9^*C*^, 0.82*^W^, 0.85^*W*^	(102), (48) [•]	
	PBEsol	0.75*^W^, 0.78^*W*^	(48) [•]	
Cs_2_AgSbCl_6_ (Indirect)	HSE	2.22*^V^, 1.49^*C*^, 2.40*^,*V*^	(61, 102, 97)	2.28^94^
	mBJ	2.23*^W^, 2.32^*W*^	(48) [•]	2.70^38^
	PBE	1.55^*C*^, 1.33*^W^, 1.40^*W*^	(102), (48) [•]	2.57^15^
	PBEsol	1.24*^W^, 1.30^*W*^	(48) [•]	
Cs_2_AgSbI_6_ (Indirect)	HSE	0.41^*C*^, 0.95*^V^	(102, 97)	
	mBJ	0.78*^W^, 0.84^*W*^	(48) [•]	
	PBE	0.44^*C*^, 0.32*^W^, 0.39^*W*^	(102), (48) [•]	
	PBEsol	0.28*^W^, 0.35^*W*^	(48) [•]	
Cs_2_AgFeCl_6_ (Direct)	PBE	1.17*^V^, 0.95*^V^	(102, 97)	
	mBJ	0.78*^W^, 0.84^*W*^	(48) [•]	
	PBE	0.44^*C*^, 0.32*^W^, 0.39^*W*^	(102), (48) [•]	
	PBEsol	0.28*^W^, 0.35^*W*^	(48) [•]	

a V, W, Q, Y, and C stand for VASP, WIEN2k, Quantum Espresso, Yambo, and CASTEP, respectively. (*) means inclusion of SOC effects. References are listed in the same order as for the respective band gap values. The symbol [•] indicates the same reference as the previous one. Exp. stands for the reported experimental values.

The overall electronic structure is consistent across different levels of theory, but differences in the band gap values are significant. For instance, it is well-known that the PBE and PBEsol functionals tend to underestimate band gap values, which can be corrected by using hybrid functionals or through GW methods. However, PBE and PBEsol are reliable for describing electronic states and band morphology while being computationally much less demanding compared to hybrid functionals. The inclusion of spin–orbit coupling (SOC) in HDP generally causes little changes in optical properties.^39,83,99,106^ The most significant effect of including SOC is reflected in the position of the electron bands, generally resulting in a small decrease in the band gap value. Due to the high computational demand, many theoretical studies on HDP only use SOC to test selected results or ignore it altogether.

Differences in the reported band gap values using the same type of functional appear to be unrelated to the software package employed and can primarily be attributed to calculation parameters such as the size of the k-grid, cutoff energies, and convergence criteria for force, pressure, and energy. Additionally, hybrid functionals are also sensitive to the value of the Hartree–Fock fraction. Using the experimental values in Table 5, hybrid functionals improve the accuracy of bandgaps predicted compared to PBE, but are computationally expensive. mBJ functionals, which use empirical potentials, do not incorporate exact exchange like hybrid functionals, and while they are computationally more efficient, they may also be less accurate. The GW method, grounded in many-body theory, is considered the most accurate for predicting bandgaps but is the most computationally demanding. In summary, mBJ, HSE functionals, and the GW method provide improved bandgap values compared to PBE-type functionals, with increased computational demands proportional to their increased accuracy.

### Alloying Effects

Due to the large indirect or direct forbidden bandgap transition in HDP, strategies involving doping, alloying, or introducing disorder are pursued to convert the indirect bandgap into a direct one or to modify the band parity, enhancing their potential for optoelectronic applications.

**Figure 5:**
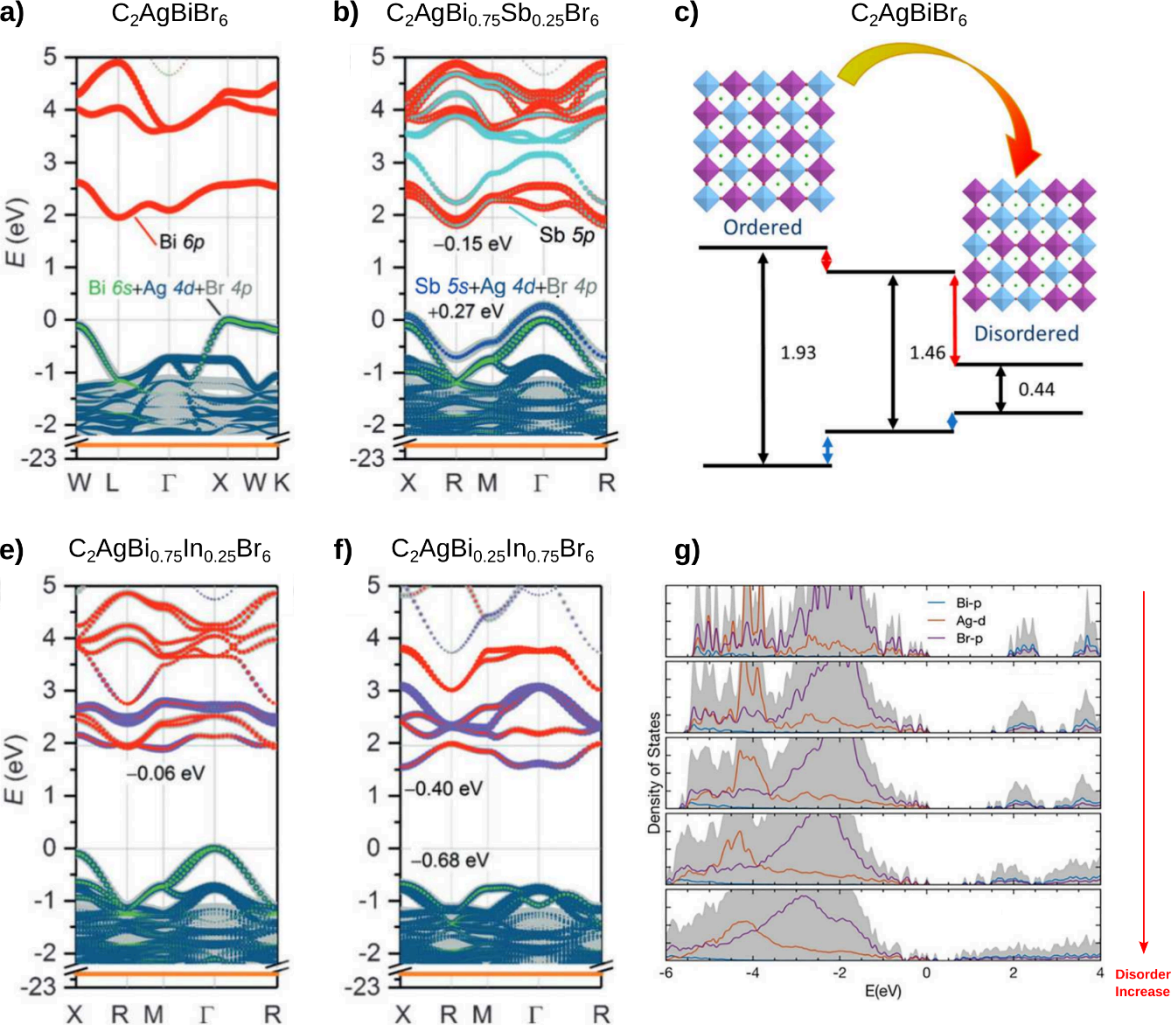
Electronic band structure of (a) Cs_2_AgBiBr_6_, (b) Cs_2_AgBi_0.75_Sb_0.25_Br_6_, (e) Cs_2_AgBi_0.75_In_0.25_Br_6_, and (f) Cs_2_AgBi_0.25_In_0.75_Br_6_. (c) Band alignment and bandgap of Cs_2_AgBiBr_6_ as a function of disorder degree: fully ordered, partially disordered, and fully disordered. (g) DOS of Cs_2_AgBiBr_6_ as a function of disorder increase. (a, b, e, f) Adapted with permission from ref (70), Copyright 2017 Wiley-VCH Verlag GmbH & Co. KGaA, Weinheim. (c) Reprinted with permission from ref (19), Copyright 2018, American Chemical Society. (g) Adapted with permission from ref (67), Copyright 2020 The Authors. Published by Wiley-VCH Verlag GmbH & Co. KGaA.

Du et al. demonstrated bandgap engineering in Cs_2_AgBiBr_6_ through In/Sb alloying on Bi site, experimentally confirming an increase in the band gap as the percentage of In increased up to 75% (Cs_2_AgBi_0.25_In_0.75_Br_6_), and a decrease in the band gap with the inclusion of Sb up to 37.5% (Cs_2_AgBi_0.625_Sb_0.375_Br_6_), with the smallest band gap value being 1.86 eV. This allowed to lower the band gap of Cs_2_AgBiBr_6_ by 0.41 eV. These trends were then studied with HSE+SOC functionals. DFT calculations show that the substitution for Sb introduces 5s states into the VBM with higher energies and 5p states into the CBM (Figure 5b) with energies similar to those in pristine Cs_2_AgBiBr_6_ (Figure 5a). These changes results in a band gap decrease, consistent with experimental results.^70^ Analogous trends with the incorporation of Sb were obtained theoretically through PBE and HSE+SOC.^107^

The degree of ordering of B-site cations in Cs_2_AgBiBr_6_ was investigated with a few different approaches, summarized in Figure 5. Substituting Bi with either In or Sb should cause the band gap to increase or decrease, respectively, in a monotonic fashion. The corresponding simulated band structure, on the other hand, shows contradictory results only for intermediate amounts of In (25%), where the CBM and the band gap were predicted to be slightly lowered instead, see Figure 5e.^70^ As a result, for heavy amounts of In, or for Sb substitution, the expected trends were otherwise found, leading to the conclusion that the complete Bi/In ordering in the supercell does not reflect the actual structure, and some kind of clustering of the [InBr6] octahedra occurs. Other authors later examined the Ag/Bi distribution in stoichiometric Cs_2_AgBiBr_6_, allowing for different local deviations from the perfect alternation. Yang et al. in ref (19), through DFT HSE+SOC calculations, demonstrated that by modifying the ordering parameters, the indirect band gap can vary from 1.93 eV for a fully ordered structure to 0.44 eV for a completely random distribution (see Figure 5c). Recently, Liu et al.^108^ studied the origin of band gap reduction in disordered Cs_2_AgBiBr_6_ using the same level of theory (HSE+SOC). They attributed this reduction to Ag/Bi disorder, which induces different Ag–Br–Ag and Bi–Br–Bi configurations, forming homoatomic clusters that cause wave function localization at the band edges. By analyzing various Ag/Bi alloy ratios, they quantified the degree of disorder and calculated the corresponding band gap reduction, reaching 0.6 eV at 25% disorder. A disorder level of 12.5% has minimal impact on the lattice constants, whereas higher alloy ratios lead to lattice expansion and structural distortion, affecting the stability of Ag-HDP. Experimentally, the degree of ordering can be modified by controlling the growth temperature of the perovskites. Ji et al. developed a crystal-engineering strategy to control the cation alternation in Cs_2_AgBiBr_6_.^67^ By using different evaporation temperatures during crystallization, the band gap was lowered by decreasing the ordering at the Ag–Bi sites. This is evident in the electronic density of states (DOS) calculated at the HSE+SOC level. As the disorder increases, defects are introduced in the Bi-p/Br-p states, progressively decreasing the conduction band, as shown in Figure 5g. While interesting from the point of view of fundamental modeling, it should also be pointed out that such a large segregation would lead to significant and perhaps unrealistic local charge imbalance.

**Figure 6:**
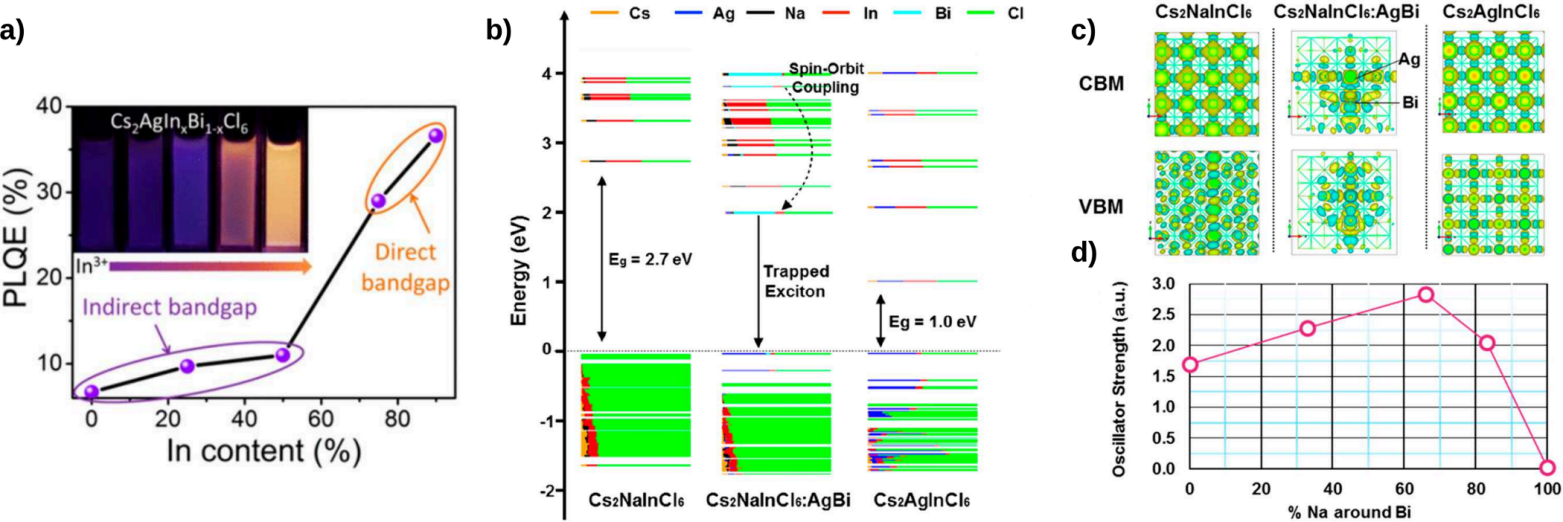
(a) Photoluminescence quantum efficiency as a function of the In content. The nature of the transition shifts from indirect to direct at high concentration of In. (b) Projected density of states for Cs_2_NaInCl_6_ (left panel), Cs_2_AgInCl_6_ (right panel), and Bi–Ag doped Cs_2_NaInCl_6_ (middle panel). Doping with Ag and Na allows the self-trapped exciton transition. (c) CBM and VBM atomic orbitals for pristine and doped perovskite. Doping with Bi–Ag localizes the wave functions. (d) Oscillator strength as a function of Na content for Bi–Ag doped Cs_2_NaInCl_6_ perovskite. (a) Reprinted with permission from ref (99), Copyright 2018, American Chemical Society. (b, c, and d) Reprinted with permission from ref (109), Copyright 2019, American Chemical Society.

Yang et al. demonstrated that the gap of the perovskite Cs_2_AgIn_*x*_Bi_1–*x*_Cl_6_ can be tuned from indirect (at low concentrations of In, *x* ≤ 0.25) to direct (at high concentrations of In, *x* > 0.75).^99^ This tuning also significantly impacts the photoluminescence quantum efficiency, increasing it by a factor of 5 (Figure 6a). However, DFT/PBE calculations indicate that the emission arises from a forbidden direct transition. Efforts to alter the forbidden direct character of double perovskites are ongoing through alternative alloying strategies. Luo et al. found that incorporating Na into the Cs_2_AgInCl_6_ perovskite modifies the parity of the electronic wave function, thereby increasing the probability of electronic transitions. By calculating the transition dipole moment (μ), they observed that the transition probability increases as the Na percentage rises to 40%, and then decreases again at higher concentrations.^17^ This aligns with their own experimental results showing that Cs_2_AgInCl_6_ with a 40% Na concentration exhibits a three-orders-of-magnitude enhancement in photoluminescence. A similar study has been conducted on Bidoped Cs_2_Ag_1–*x*_Na_*x*_InCl_6_ by Locardi et al. They found that photoluminescence was enhanced for Bi and Na concentrations of 5% and 60%, respectively. Through DFT/PBE level calculations for a 3% Bidoped concentration, they analyzed the DOS, showing that Ag and Bi ions in the Na-rich system act as localization centers for electrons and holes at the band edge (middle section of Figure 6b). Analysis of the atomic orbitals for the CBM and VBM shows that the wave functions are delocalized in the pristine perovskite (left and right panels of Figure 6c). However, doping with Ag and Bi causes the wave functions to become localized (middle section of Figure 6c), allowing efficient trapped exciton emission. Similar to the previous work by Juo et al., they found that the oscillator strength, or dipole moment, increased up to a Na percentage of 66% and then decreased (Figure 6d). The results align with the experimental findings, where the photoluminescence quantum yield increased from 6% to 22% up to a Na concentration of 60% and then decreased. Such behavior corresponds to the strong localization of the hole wave function near the Ag center, which disappears for considerable Na concentrations (>66%).^109^ More recently, a study combining Tight Binding and DFT found similar trends in the transition probability for Cs_2_AgInCl_6_ when alloyed with Na.^23^

**Figure 7:**
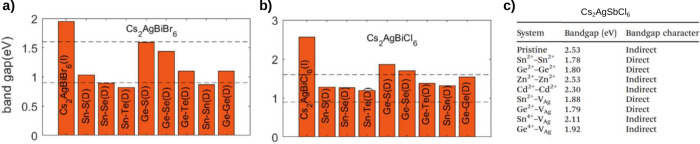
DFT/HSE+SOC bandgaps for divalent doping in (a) Cs_2_AgBiBr_6_, (b) Cs_2_AgBiCl_6_, and (c) CsAg_2_SbCl_6_. In a)-b), the nature of the transition, Direct or Indirect, is indicated by (D) or (I), respectively. In (c), V_Ag_ stands for Ag vacancy. (a and b) Adapted with permission from ref (80), Copyright 2021, American Chemical Society. (c) Adapted with permission of Royal Society of Chemistry, from ref^62^, Copyright 2022; permission conveyed through Copyright Clearance Center, Inc.

Other strategies to change the transition character from indirect to direct in double perovskites have been explored theoretically through doping with divalent cations like Sn^2+^ and Ge^2+^ in Cs_2_AgBiCl_6_, Cs_2_AgBiBr_6_,^80^ and Cs_2_AgSbCl_6_.^62^ By replacing one monovalent and one trivalent cation with two divalent cations, the charge neutrality is maintained, and at a doping concentration of 25% this was predicted to be energetically favorable by using formation energy and decomposition enthalpy calculations at the DFT/HSE+SOC level. The predicted bandgap values, ranging from 0.9 to 2.5 eV (Figure 7), are suitable for photovoltaic applications, while the optical absorption was enhanced by this kind of doping. On the other hand, Fe alloying in Cs_2_AgBi(Cl,Br)_6_ perovskites has been studied and synthesized, demonstrating a band gap transition from indirect to direct. Fe incorporation lowers and restructures the CBM, enabling band gap tunability in the range of 1 to 2.5 eV and enhancing the photoluminescence quantum yield. These properties highlight the potential applications of these materials in photoelectronic and photovoltaic devices.^74,75^ These studies provide valuable guidance for the future electronic structure engineering of double perovskites. Nonetheless, given that photoluminescence in HDP is generally low, further investigations are required to evaluate the transition probabilities of the reported direct gap compounds and explore methods for their improvement. Furthermore, since many of these studies are focused on cubic symmetries, it is crucial to consider potential transitions to more stable nonperovskite phases and the strategies to suppress such transitions in order to enhance stability.

Altogether, bandgap engineering through alloying at the cation and anion sites of double perovskites has been extensively studied using theoretical methods in various perovskite materials. These studies enhance the understanding of the fundamental properties of these materials and offer valuable guidance for experimental syntheses, frequently demonstrating strong agreement. In Table 6, we summarize the band gaps reported for alloying at the monovalent or trivalent cation site of Ag-based HDP. One might expect the position of the bands due to cation doping follow a linear behavior, however, there are several examples where the so-called Bandgap Bowing Effect is observed. In fact, in Table 6, we can see that in most materials and methods, the band gap decreases and then increases again as the value of “*x*” increases. This nonlinear band gap change in HDP is an open topic, and its causes are still under discussion.^88,112^ Factors potentially related to this effect include changes in lattice parameters, octahedral distortion, chemical effects that modify the positions of the band states, and ordering. Gupta et al. found a positive correlation between chemical effects, lattice expansion, and bandgap bowing, while octahedral distortion and lattice compression reduce the bowing effect in both Cs_2_Ag_*x*_Na_1–*x*_InCl_6_ and Cs_2_AgIn_*x*_Bi_1–*x*_Cl_6_.^23^

**Table 6:** Band Gap Values, in Units of eV, for Monovalent and Trivalent Alloying in Ag-Based Perovskites^a^

	*x*						
Perovskite	0.00	0.25	0.50	0.75	1.00	Method	Ref.
Cs_2_(Ag_*x*_Na_1–*x*_)InCl_6_	5.31^*D*^	3.72^*D*^	3.07^*D*^	3.18 ^*D*^	3.29^*D*^	TB	(23)
Cs_2_(Ag_*x*_Cu_1–*x*_)SbCl_6_	—	—	1.8	1.88	2.22	HSE	(61)
Cs_2_Ag(In_*x*_Bi_1–*x*_)Cl_6_	2.97	2.49	2.32	2.85^*D*^	3.27^*D*^	TB	(23)
Cs_2_Ag(In_*x*_Bi_1–*x*_)Br_6_	2.0	1.94	—	2.28 ^*D*^	1.32 ^*D*^	HSE*	(70)
Cs_2_Ag(Sb_*x*_Bi_1–*x*_)Cl_6_	1.83	1.54	1.42	1.38	1.37	PBE	(107)
	1.53	1.20	1.09	1.01	1.34	PBE*	
	3.09	2.66	2.51	2.47	2.46	HSE	
	2.72	—	2.10	—	2.37	HSE*	
Cs_2_Ag(Sb_*x*_Bi_1–*x*_)Br_6_	1.77	1.18	0.94	0.82	1.37	HSE*	(88)
	2.00	1.58	—	—	1.67	HSE*	(70)

	*x*						
Perovskite	0.00	0.30	0.50	0.80	1.00	Method	Ref.
Cs_2_Ag(Sb_*x*_Bi_1–*x*_)Br_6_	1.32	1.26	1.14	1.04	0.89	PBE	(110)
	1.10	1.08	1.03	0.95	0.85	PBE*	
	2.17	2.09	1.96	1.85	1.69	TB09	
	1.95	1.91	1.85	1.76	1.65	TB09*	

a Most gaps are indirect, except for those marked with the superscript *D*, which are direct. The values from ref (110) were calculated using ABINIT.^25^ The TB09 method^111^ includes PBE-type functionals. All of the other values are calculated using VASP. (*) indicates that SOC effects are included.

Another interesting effect occurs when trivalent cations such as In and Bi are mixed, which give rise to type I (direct) or type II (indirect) materials on their own (Table 6). The indirect character is maintained up to percentages below 75% of the type I cation. When the 75% of the type I cation is exceeded, the delocalized states in the conduction band lower in energy, which changes the nature of the transition from indirect to direct. This effect, also observed experimentally,^99^ enables the conversion of the indirect transitions to direct through trivalent cation doping, enhancing the photoluminescence quantum yield.

In order to compare the differences between doping at the cation and anion sites, in Table 7 we present the reported band gaps for alloying at the anion site. The bowing effect is absent, and linear behavior is observed in the band gap modification. As “x” increases, the band gap increases linearly. This suppression of the bowing effect may be due to the fact that the ionic radius of Cl (1.81 Å in VI coordination) is slightly smaller than that of Br (1.96 Å), causing the two previously mentioned effects to act as suppressors: (i) there is a lattice parameter compression as the Cl percentage increases and (ii) by alloying the two types of anions, Cl–Br, the octahedra in the material exhibit greater distortion.

**Table 7:** Band Gap Values for Anion Alloying in Ag-Based Perovskites^a^

	Cs_2_AgInBr_6–*x*_Cl_*x*_			Cs_2_AgSbBr_6–*x*_Cl_*x*_		
*x*	(D) Gap (eV)	Method	Ref.	(I) Gap (eV)	Method	Ref.
*x* = 0	1.64	HSE	98	1.68	HSE	(20)
				1.73	G_0_W_0_	
				1.77	PBE0	(60)
*x* = 1	1.92	HSE	98	1.82	HSE	(20)
				1.90	G_0_W_0_	
				1.87	PBE0	(60)
*x* = 2	2.16	HSE	98	1.87	HSE	(20)
				1.98	G_0_W_0_	
				1.96	PBE0	(60)
*x* = 3	2.32	HSE	98	2.01	HSE	(20)
				2.13	G_0_W_0_	
				2.22	PBE0	(60)
*x* = 4	2.43	HSE	98	2.17	HSE	(20)
				2.28	G_0_W_0_	
				2.31	PBE0	(60)
*x* = 5	2.63	HSE	98	2.29	HSE	(20)
				2.41	G_0_W_0_	
				2.43	PBE0	(60)
*x* = 6	3.00	HSE	98	2.34	HSE	(20)
				2.49	G_0_W_0_	
				2.72	PBE0	(60)

a (D) and (I) stand for direct and indirect gaps, respectively. HSE and G_0_W_0_ calculations carried out with VASP, and PBE0 calculations carried out with Crystal17.^113^

### Effect of Pressure

Band gap engineering was observed in double perovskite materials through applied pressure. Pressure was shown to have a direct relationship with changes in excitonic absorption and band gap quenching in cubic perovskites attributed to octahedral contractions and a decrease in atomic bond distances. These changes result in the proximity and overlap of atomic orbitals, causing a closer alignment of the VBM and CBM.^3,73,114−116^

**Figure 8:**
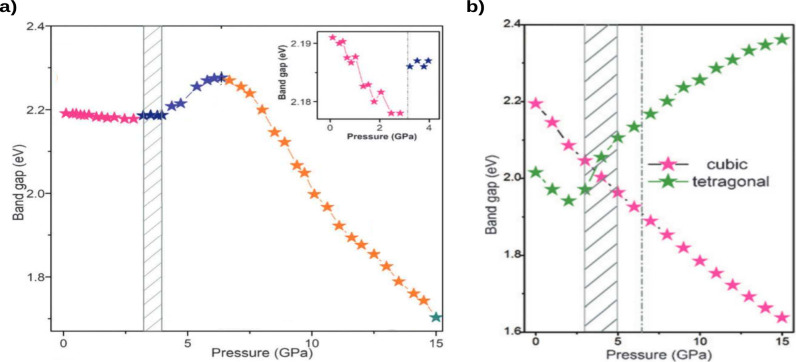
Pressure-induced modifications of HDP. (a) Experimental band gap of Cs_2_AgBiBr_6_ as a function of pressure. The inset provides a zoom on the range from 0 to 4 GPa, where a cubic-to-tetragonal phase transition occurs. The colors differentiate changes in the trends. (b) DFT-calculated band gaps for the cubic and tetragonal phases of Cs_2_AgBiBr_6_ as a function of pressure. (a and b) Adapted with permission from ref (116), Copyright 2017 Wiley-VCH Verlag GmbH & Co. KGaA, Weinheim.

Li et al. reported one of the first high-pressure treatment on double perovskites, lowering the band gap of Cs_2_AgBiBr_6_ from 2.2 to 1.7 eV under pressures up to 15 GPa (see Figure 8a).^116^ A phase transition from cubic  to tetragonal (*I*4/*m*) was observed. The cubic-tetragonal phase combination caused a nonlinear change in the band gap over specific pressure ranges, as shown in Figure 8a. Notably, after releasing the pressure, the band gap is partially retained at a value of 2.0 eV. Despite the ability to reduce the band gap, its nature remains indirect and its value is still considerably high for photovoltaic applications. In line with these findings, Fu et al. reported on the pressure-induced effects in Cs_2_AgBiBr_6_ nanocrystals. They also reported, a phase transition from cubic to tetragonal at pressures of 2.3 GPa.^117^ Due to this phase transition, it was noted that as pressure increases, the band gap first decreases before increasing again. Through DFT calculations, both cubic and tetragonal phases were studied as functions of pressure. It was found that the band gap in the cubic phase decreases as pressure increases (from 0 to 15 GPa), while for the tetragonal phase, the gap decreases until reaching 3 GPa, after which it begins to increase (Figure 8b). The evolution of the band gap for both phases is attributed to modifications in orbital interactions resulting from the tilting and distortion of the Ag–Br and Bi–Br octahedra under pressure. These results are consistent with experimental evidence, which indicates that up to 2.3 GPa, the perovskite phase remains cubic, exhibiting a decrease in the band gap. Beyond this pressure, a phase transition occurs from cubic to tetragonal, resulting in an increase in the band gap.

Several studies have calculated the effects of pressure using DFT for cubic perovskites, including Cs_2_AgBi(Br,Cl)_6_,^115^ Cs_2_AgSbCl_6_,^73^ and Cs_2_(Ag,Na)In(Bi,Sb)Cl_6_ alloys.^118^ A general observation from these studies is that pressure induces a decrease in the band gap. However, as previously described, phase changes may occur, resulting in the opposite trend. Some syntheses of cubic perovskites report considerably lower band gap values compared with typical ones, which are attributed to strain effects. For example, the Cs_2_AgSbCl_6_ perovskite has been reported to have the lowest band gap value of 1.82 eV, which is because of pressure effects on its structure.^73^ DFT calculations confirmed that a reduction in the lattice parameter of the Cs_2_AgSbCl_6_ perovskite results in a decrease in the band gap.

So far, we discussed various examples where doping modifies the optical properties of HDP, with these changes attributed to the various electronic states introduced by the dopants. In fact, ionic sizes can also influence properties through strain engineering. Shaek et al. identified shifts in the emission peak of Cs_2_Ag_0.4_Na_0.6_In_1–*x*_Bi_*x*_Cl_6_ with varying Bi doping concentrations, while increased Sb concentration in Cs_2_NaIn_1–*x*_Sb_*x*_Cl_6_ does not result in appreciable changes.^118^ These behaviors were linked to the electronic properties and ionic sizes of the dopants. While Bi^3+^ is significantly larger than In^3+^ , Sb^3+^ has a very similar ionic radius. The strain induced by Bi substitution indicates that increasing its concentration generates greater strain, resulting in a lower bandgap. Conversely, Sb induces only minor strain, which in turn does not shift the bandgap. DFT calculations reflect an increase in the lattice parameter with higher Bi concentrations, consistent with experimental lattice spacing and the introduction of strain into the system.

### Dielectric Function

The optical features of double perovskites determine their possible applications in optoelectronics and/or photovoltaics. These are typically evaluated through the complex dielectric function *ε*(ω) and the absorption coefficient α(ω). The complex dielectric function
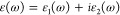
11

is a fundamental property that defines the interaction of a material with electromagnetic waves. *ε*(ω) is composed of the real part *ε*_1_(ω), which is associated with polarization and refraction, and the imaginary part, *ε*_2_(ω), which is related with the possible interband transitions and captures the material’s light absorption. Of particular interest is the static dielectric constant, defined as the real dielectric function at zero frequency, *ε*_1_(0). This indicates the polarization response to a static electric field, and is correlated with excitonic binding energy^119^ and charge mobility. Higher values of *ε*_1_(0) correspond to enhanced charge mobility,^120^ and improved device performances.^20,121^

The absorption coefficient indicates the amount of light that can be absorbed by the material. It is calculated from the complex dielectric function as follows:
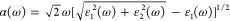
12

**Figure 9:**
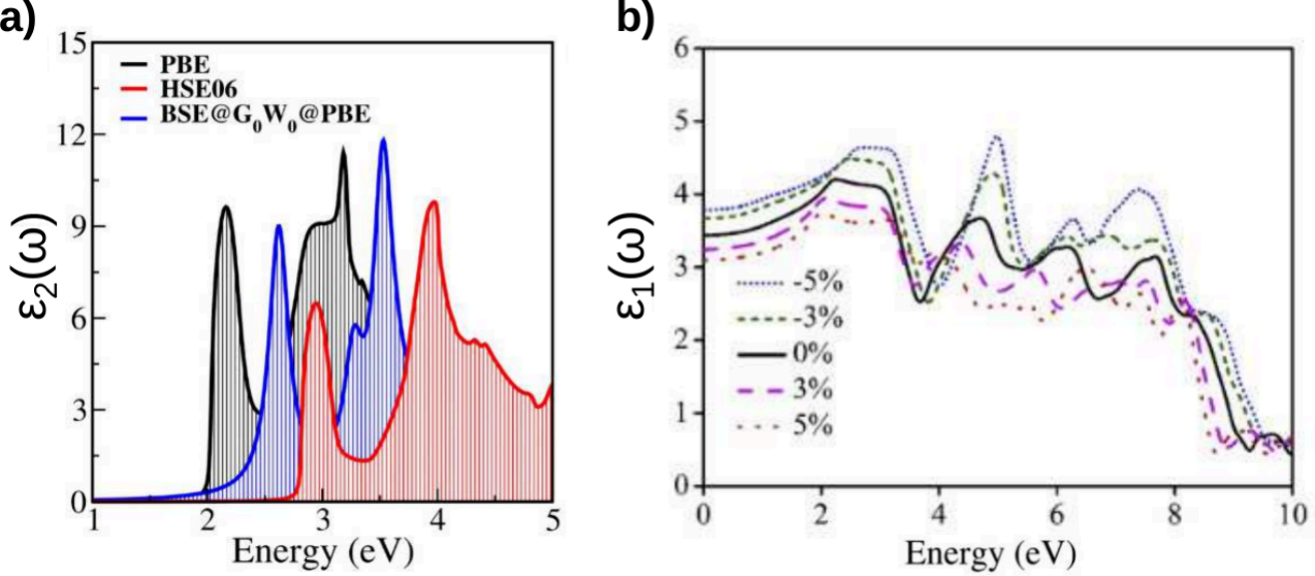
(a) Complex dielectric function of Cs_2_AgSbBr_6_ calculated using the PBE, HSE, and BSE-GW-PBE methods. (b) Effects of strain on the real dielectric function of Cs_2_AgInCl_6_; different line styles and colors indicate the percentage of strain applied. (a) Adapted with permission from ref (20), Copyright 2024 by the American Physical Society. (b) Reprinted with permission from ref (106), Copyright 2019 Elsevier B.V. All rights reserved.

Similarly, other optical properties, such as refractive index η(ω) and reflectivity *R*(ω), can be derived from the complex dielectric function. Various methods are employed to accurately calculate the dielectric function in double perovskites, including GGA or hybrid functionals, and many-body perturbation theory (MBPT-BSE@GW@PBE).^32^ The main difference between these methods lies in the estimation of the band edge, leading to different peak positions of the dielectric functions and values for the static dielectric constants. In Figure 9a the absorption, *ε*_2_(ω), calculated using different methods for the perovskite Cs_2_AgSbBr_6_ is shown. The position of the band edge, the intercept along the energy axis in the plot of the imaginary dielectric function, varies depending on the method used, but the shape of the function exhibits a certain degree of similarity. The static dielectric constant was also calculated, with differences up to 20% depending on the method employed.^110^ Although the absolute values can vary considerably depending on the calculation approach, most methods show agreement in terms of trends and changes. For example, consistent trends were observed in the increase of the static dielectric constant with increasing values of ‘*x*’ in Cs_2_AgSb_*x*_Bi_1–*x*_Cl_6_ across three different theoretical methods.^110^

Interestingly, most theoretical methods agree that Ag-based perovskites exhibit an adequate range of absorption from the calculated dielectric function, primarily within the visible light spectrum and extending into the UV range. Additionally, all calculations predict a high absorption coefficient in Ag-based perovskites, on the order of 10^5^ cm^–1^, indicating high-intensity optical transitions at the band edge that increase rapidly for higher energy values (refs (20, 40, 48, 80, 96, 102, 106, 107, 110, 122, and 123)). Despite all these favorable predictions, the static dielectric constants of Ag-based perovskites are all relatively low, ranging between 2 and 5, making them less suitable for single-junction solar cells compared with their Pb-based counterparts. However, strategies such as doping or pressure-induced effects can enhance the static dielectric constant. In Figure 9b, it is shown how the real part of the dielectric function is modified by strain, thereby affecting the static dielectric constant. All in all, these materials exhibit a combination of versatility and tunability, making them especially promising for advanced applications, such as photodetectors, visible LEDs, lasers, and UV shielding devices.

## Functional Comparison

In this section, we present a highly summarized overview of the functionals used by DFT in the study of HDP properties. There are many other functionals in the literature that can be categorized within the types presented in Table 8.

**Table 8:** Comparison of DFT functionals for halide double perovskites

Functional	Type	Decomposition and Formation Energy	DOS, Bands, and Bandgap	Dielectric Function	Cost
PBE/PBEsol	GGA	Fair	Underestimated bandgap,	Overestimated	Low
		May overestimate stability	Good qualitative electronic structure		
HSE06/ PBE0	Hybrid	More accurate than PBE	Improved valence, and conduction bands, more accurate gap	More accurate than PBE	High
GW	Beyond DFT	(from PBE/HSE06)	Precise electronic structure, gap close to experiment	Best optical description	Very High

## Conclusions

This review gives an outline of the recent significant advancements in the theoretical study of the structural and optical properties of Ag-based HDP through *ab initio* calculations. DFT has proven essential not only in elucidating the fundamental mechanisms that govern the stability, electronic structure, and optical properties of these materials but also in proposing methods to manipulate and enhance these properties. Ag-based HDP have garnered considerable attention as nontoxic, stable alternatives to lead halide perovskites for optoelectronic applications, and their full potential can be achieved with the help of detailed theoretical and computational analysis that complements experimental research.

To capture the complex behavior of HDP accurately, a variety of DFT software packages have been employed by the scientific community, including VASP, ABINIT, WIEN2k, CASTEP, CRYSTAL, and Quantum Espresso. Each package offers advantages depending on the implementation and optimization of computational methods, system size, and level of precision needed for specific properties. While the input parameters chosen for DFT calculations can vary slightly from one software to another or even within the same software, the general trends show substantial agreement across different packages. In fact, when comparing results, regardless of the software used, the most critical point is the choice of functional. While functionals with the local density approximation (LDA) are the simplest and easiest to implement, they come with significant limitations and limited accuracy. More computationally expensive functionals, with the implementation of the generalized gradient approximation (GGA), such as PBE or PBEsol, reproduce the electronic properties of HDP more accurately but tend to underestimate values such as band gaps or dielectric constants. These functionals are widely used anyway to study thermodynamic stability with reliable results and little computational cost when compared to more precise methods. They also provide an accurate description of the HDP structures, e.g., lattice parameters, electronic density of states, charge densities, and chemical bonding, among others. Hybrid functionals have been used to estimate the electronic properties more accurately but at a higher computational cost. Finally, many-body perturbation theory (in the GW approximation) also improves the accuracy of the electronic and optical predictions, yielding property values that align more closely with experimental measurements. However, also this is computationally expensive and may not be feasible for larger systems or systematic defect analyses. Overall, the choice of functional depends on the target accuracy and available computational resources.

The geometric tolerance factors (i.e., Goldschmidt’s tolerance factor, octahedral factor, and τ factor) provide an indication of how close materials are to the ideal perovskite structure “elpasolite” and, consequently, their overall stability. These factors have served as inputs for theoretical methods, whether DFT, ML, or a combination of both, to narrow down the vast number of materials to those with the most promising optoelectronic properties. Several of these materials were first proposed theoretically and were later successfully synthesized and characterized, as seen in the case of Ag–Bi, In, and Sb HDP. Beyond the first approximation based on tolerance factors, thermodynamic analysis is necessary to determine stability. The formation energy from elements provides a necessary condition of stability during the synthesis; then, the decomposition enthalpy provides a second condition of stability toward decomposition into ternary and binary materials: these latter reactions have direct correlates in the degradation observed often in halide perovskites. General agreement between DFT thermodynamic predictions and experimental synthesis outcomes has been found. Cases such as Cs_2_AgBiBr_6_, Cs_2_AgSbCl_6_, and alloys of Cs_2_AgInBr_6_, Cs_2_AgSbBr_6_, and Cs_2_AgBiCl_6_ were first predicted based on formation energy calculations and were later successfully synthesized. In turn, decomposition enthalpy calculations have helped us to understand the degradation mechanisms of HDP, as evidenced in the case of Ag HDP into Cs–Ag-halide and Cs-*B*‴-halide compounds. Iodide Ag-HDP, where both the tolerance factor *t* and the τ factor indicate a significant deviation from unity, the calculated formation energy is high compared to other HDP, and its decomposition enthalpy reveals highly probable degradation pathways: all of these aspects align with the difficulty in their synthesis. DFT calculations have also been useful for studying defect formation and for proposing experimental strategies to suppress them. In the case of Ag-HDP, one of the most probable defects identified is *B*‴ vacancies, along with Ag/*B*‴ antisites. Halogen-*B*‴ poor or rich environments have been proposed and employed, resulting in the synthesis of more stable HDP. In this way, one can conclude that *ab initio* thermodynamics calculations provide a deep understanding of the stability of perovskites, not only when evaluating the pure phases but also with respect to defect formation and alloying of different elements.

Theoretical studies have also provided insights into the formation of defects and alloying and into the role of vacancies, dopants, and cation–anion disorder in tuning the optical properties of HDP. DFT calculations show how doping strategies can alter the indirect nature of the optical transitions in HDP, change the parity of the transition to enable direct transitions, and increase the oscillator strength or dipole moment through alloying. This has been experimentally observed through Na doping in Cs_2_AgInCl_6_ and In doping in Cs_2_AgBiCl_6_, where the transition is modulated from indirect to direct, increasing the quantum efficiency of the perovskites. The degree of disorder of the B-site cations plays a fundamental role in tuning the band gap and the overall stability. DFT calculations estimated band gap variations of up to 1.49 eV for a fully random cation-ordered Cs_2_AgBiBr_6_ HDP. This finding motivated the investigation of synthesis techniques, which demonstrated that controlling the vaporization temperature during crystallization modified the degree of cation disorder, achieving band gap reductions of up to 0.26 eV. Additionally, variations in the band gap of the HDP, whether due to the defect strategies, or pressure-induced effects, align with experimental observations, as seen in the case of Cs_2_AgBiCl_6_ and Cs_2_AgBiBr_6_ perovskites.

In conclusion, DFT methods provide a comprehensive framework for understanding, predicting, and optimizing the stability and electronic and optical properties of Ag-based HDP, offering valuable guidance for experimental efforts. For their part, Ag-based HDP has demonstrated high stability, allowing for the inclusion of defects and doping, which facilitates the manipulation and enhancement of their properties, making them an attractive material with potential for high-performance applications. The refinement of DFT methodologies in exchange-correlation functionals, incorporating machine learning methods, optimizing computational resources through GPU parallelization, and the exploration of Ag-HDP and diverse compositions, particularly through materials and defect engineering, will pave the way for sustainable, nontoxic materials for optoelectronic applications.
